# Tri­aqua­(pyrazole-4-carboxyl­ato-κ*N* ^1^)lithium

**DOI:** 10.1107/S160053681301831X

**Published:** 2013-07-06

**Authors:** Wojciech Starosta, Janusz Leciejewicz

**Affiliations:** a Institute of Nuclear Chemistry and Technology, ul. Dorodna 16, 03-195 Warszawa, Poland

## Abstract

In the monomeric title complex, [Li(C_4_H_3_N_2_O_2_)(H_2_O)_3_], the Li^+^ cation is coordinated by a pyrazole N atom and three water mol­ecules in a distorted tetra­hedral geometry. The carboxyl­ate group is deprotonated. The complex mol­ecules are involved in O—H⋯O and N—H⋯O hydrogen bonding, forming layers stacked along the *b* axis.

## Related literature

For the structure of pyrazole-4-carb­oxy­lic acid, see: Foces-Foces *et al.* (2001 ▶).
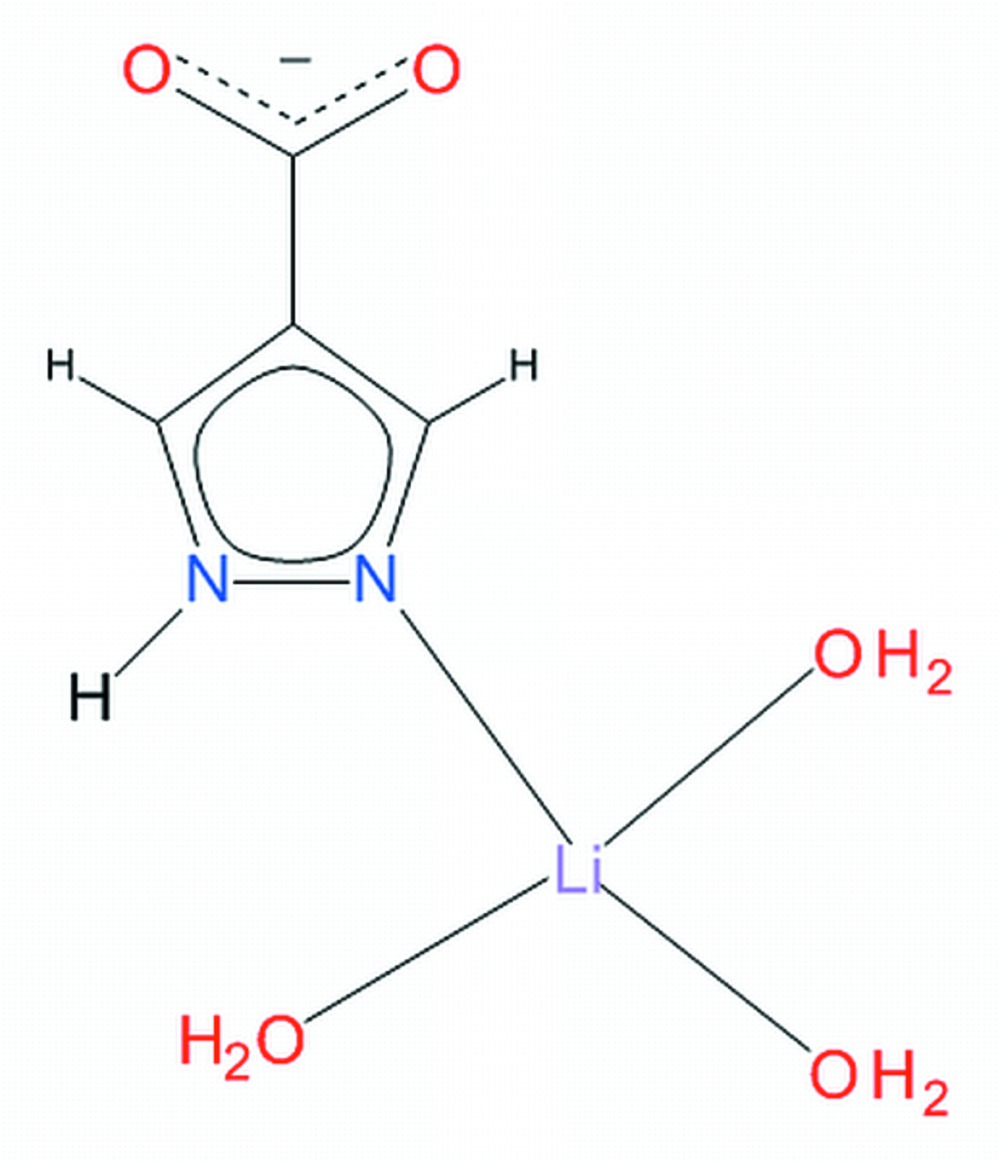


## Experimental

### 

#### Crystal data

[Li(C_4_H_3_N_2_O_2_)(H_2_O)_3_]
*M* *_r_* = 172.07
Orthorhombic, 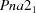

*a* = 7.2817 (15) Å
*b* = 6.9635 (14) Å
*c* = 15.186 (3) Å
*V* = 770.0 (3) Å^3^
*Z* = 4
Mo *K*α radiation
μ = 0.13 mm^−1^
*T* = 293 K
0.25 × 0.18 × 0.12 mm

#### Data collection

Kuma KM4 four-circle diffractometer
Absorption correction: analytical (*CrysAlis RED*; Oxford Diffraction, 2008 ▶) *T*_min_ = 0.967, *T*_max_ = 0.983
1161 measured reflections
1161 independent reflections
901 reflections with *I* > 2σ(*I*)
3 standard reflections every 200 reflections intensity decay: 5.9%

#### Refinement

*R*[*F* ^2^ > 2σ(*F* ^2^)] = 0.042
*wR*(*F* ^2^) = 0.119
*S* = 1.01
1161 reflections
133 parameters
7 restraints
H atoms treated by a mixture of independent and constrained refinement
Δρ_max_ = 0.34 e Å^−3^
Δρ_min_ = −0.44 e Å^−3^

### 

Data collection: *KM-4 Software* (Kuma, 1996 ▶); cell refinement: *KM-4 Software*; data reduction: *DATAPROC* (Kuma, 2001 ▶); program(s) used to solve structure: *SHELXS97* (Sheldrick, 2008 ▶); program(s) used to refine structure: *SHELXL97* (Sheldrick, 2008 ▶); molecular graphics: *SHELXTL* (Sheldrick, 2008 ▶); software used to prepare material for publication: *SHELXTL*.

## Supplementary Material

Crystal structure: contains datablock(s) I, global. DOI: 10.1107/S160053681301831X/kp2455sup1.cif

Structure factors: contains datablock(s) I. DOI: 10.1107/S160053681301831X/kp2455Isup2.hkl

Additional supplementary materials:  crystallographic information; 3D view; checkCIF report

## Figures and Tables

**Table 1:** Selected bond lengths (Å)

Li1—N2	2.053 (5)
Li1—O3	1.905 (5)
Li1—O5	1.909 (4)
Li1—O4	1.960 (5)

**Table 2:** Hydrogen-bond geometry (Å, °)

*D*—H⋯*A*	*D*—H	H⋯*A*	*D*⋯*A*	*D*—H⋯*A*
O3—H32⋯O1^i^	0.84 (2)	1.87 (3)	2.666 (3)	159 (4)
O4—H41⋯O2^ii^	0.81 (2)	1.88 (2)	2.684 (2)	171 (4)
O5—H52⋯O2^iii^	0.80 (2)	2.04 (3)	2.813 (3)	163 (5)
O4—H42⋯O2^iv^	0.84 (2)	1.94 (2)	2.770 (3)	174 (4)
O3—H31⋯O4^v^	0.82 (2)	2.05 (3)	2.820 (3)	156 (4)
O5—H51⋯O3^vi^	0.83 (2)	1.98 (2)	2.804 (3)	172 (4)
N1—H1⋯O1^i^	0.86	1.96	2.776 (3)	159

Symmetry codes: (i) 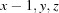; (ii) 
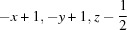
; (iii) 
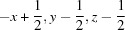
; (iv) 
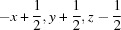
; (v) 
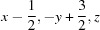
; (vi) 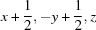.

**Fig. 1.**
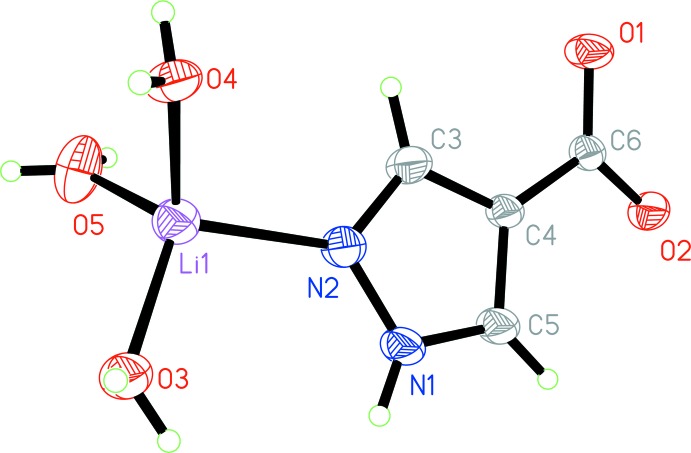
A molecule of the title complex with atom labelling scheme and 50% probability displacement ellipsoids.

**Fig. 2.**
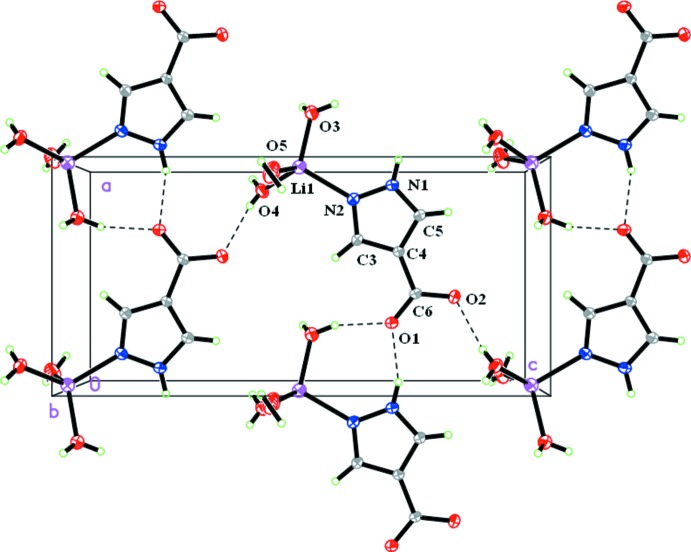
A single molecular layer viewed along the b axis.

**Fig. 3.**
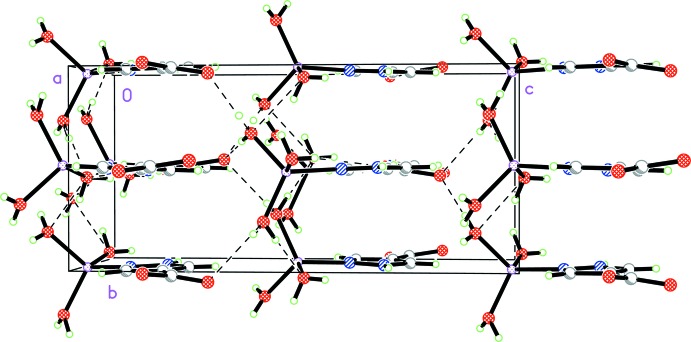
Molecular layers as viewed along the a direction.

## supplementary crystallographic information

### Comment

The orthorhombic structure of the title compound is composed of dicrete mononuclear molecules in which Li^1+^ is coordinated by the non-protonated hetero-ring N atom of the ligand molecule and three aqua O atoms at the apices of distorted tetrahedron. The observed Li—O and Li—N bond distances and bond angles reveal usual values (Table 2). The carboxylic group is deprotonated. It makes a dihedral angle of 10.7 (2)° with the almost planar [r.m.s.0.0014 (1) Å] pyrazole ring. Bond distances and bond angles within the latter are close to those observed in the structure of the parent acid (Foces-Foces *et al.*, 2001). Complex molecules form layers parallel to the unit cell *ac* plane (Fig.2) and are stacked along the *b* axis (Fig.3). Coordinated water molecules are active as donors and acceptors in an extended hydrogen bond system in which carboxylate O atoms are as acceptors (Table 3). The protonated hetero-ring N atom is as a donor and a carboxylate O atom scts as an acceptor is also observed.

### Experimental

The compound was synthetized using 1 mmol of LiOH (Aldrich) and a small excess over 1 mmol of pyrazole-4-carboxylic acid (Aldrich) dissolved in 50 mL of doubly distilled water. The solution was refluxed with stirring for 5 h and then left to crystallize at room temperature. Colourless single-crystal blocks deposited after a week. They were washed with cold ethanol and dried in the air.

### Refinement

Hydrogen atoms attached to the water molecules and protonated hetero-ring N atom were located in a difference map and refined isotropically. Two H atoms attached to pyrazole C atoms were located at calculated positions and treated as riding on the parent atoms with C—H=0.93 Å.

### Crystal data

**Table:**

[Li(C_4_H_3_N_2_O_2_)(H_2_O)_3_]	*F*(000) = 360
*M**_r_* = 172.07	*D*_x_ = 1.484 Mg m^−^^3^
Orthorhombic, *P**n**a*2_1_	Mo *K*α radiation, λ = 0.71073 Å
Hall symbol: P 2c -2n	Cell parameters from 25 reflections
*a* = 7.2817 (15) Å	θ = 6–15°
*b* = 6.9635 (14) Å	µ = 0.13 mm^−^^1^
*c* = 15.186 (3) Å	*T* = 293 K
*V* = 770.0 (3) Å^3^	Block, colourless
*Z* = 4	0.25 × 0.18 × 0.12 mm

### Data collection

**Table:**

Kuma KM4 four-circle diffractometer	901 reflections with *I* > 2σ(*I*)
Radiation source: fine-focus sealed tube	*R*_int_ = 0.000
Graphite monochromator	θ_max_ = 30.1°, θ_min_ = 2.7°
profile data from ω/2θ scan	*h* = 0→10
Absorption correction: analytical (*CrysAlis RED*; Oxford Diffraction, 2008)	*k* = 0→9
*T*_min_ = 0.967, *T*_max_ = 0.983	*l* = 0→21
1161 measured reflections	3 standard reflections every 200 reflections
1161 independent reflections	intensity decay: 5.9%

### Refinement

**Table:**

Refinement on *F*^2^	Primary atom site location: structure-invariant direct methods
Least-squares matrix: full	Secondary atom site location: difference Fourier map
*R*[*F*^2^ > 2σ(*F*^2^)] = 0.042	Hydrogen site location: inferred from neighbouring sites
*wR*(*F*^2^) = 0.119	H atoms treated by a mixture of independent and constrained refinement
*S* = 1.01	*w* = 1/[σ^2^(*F*_o_^2^) + (0.0972*P*)^2^] where *P* = (*F*_o_^2^ + 2*F*_c_^2^)/3
1161 reflections	(Δ/σ)_max_ < 0.001
133 parameters	Δρ_max_ = 0.34 e Å^−^^3^
7 restraints	Δρ_min_ = −0.44 e Å^−^^3^

### Special details

**Table:**

Geometry. All e.s.d.'s (except the e.s.d. in the dihedral angle between two l.s. planes) are estimated using the full covariance matrix. The cell e.s.d.'s are taken into account individually in the estimation of e.s.d.'s in distances, angles and torsion angles; correlations between e.s.d.'s in cell parameters are only used when they are defined by crystal symmetry. An approximate (isotropic) treatment of cell e.s.d.'s is used for estimating e.s.d.'s involving l.s. planes.
Refinement. Refinement of *F*^2^ against ALL reflections. The weighted *R*-factor *wR* and goodness of fit *S* are based on *F*^2^, conventional *R*-factors *R* are based on *F*, with *F* set to zero for negative *F*^2^. The threshold expression of *F*^2^ > σ(*F*^2^) is used only for calculating *R*-factors(gt) *etc*. and is not relevant to the choice of reflections for refinement. *R*-factors based on *F*^2^ are statistically about twice as large as those based on *F*, and *R*- factors based on ALL data will be even larger.

### Fractional atomic coordinates and isotropic or equivalent isotropic displacement parameters (Å^2^)

**Table:**

	*x*	*y*	*z*	*U*_iso_*/*U*_eq_	
O2	0.5914 (2)	0.4578 (3)	0.32056 (11)	0.0290 (4)	
O1	0.7088 (2)	0.5037 (3)	0.18706 (12)	0.0332 (4)	
N2	0.1533 (3)	0.4966 (3)	0.10494 (15)	0.0333 (5)	
C4	0.3883 (2)	0.4946 (3)	0.20198 (14)	0.0217 (4)	
N1	0.0900 (2)	0.5047 (3)	0.18846 (14)	0.0287 (4)	
H1	−0.0246	0.5103	0.2019	0.034*	
C6	0.5766 (3)	0.4856 (3)	0.23794 (14)	0.0214 (4)	
C3	0.3351 (3)	0.4911 (4)	0.11354 (15)	0.0335 (5)	
H3	0.4167	0.4856	0.0665	0.040*	
C5	0.2250 (3)	0.5030 (3)	0.24788 (17)	0.0281 (4)	
H5	0.2115	0.5067	0.3088	0.034*	
Li1	0.0107 (6)	0.4846 (6)	−0.0115 (3)	0.0300 (8)	
O4	0.1074 (2)	0.6710 (3)	−0.09637 (13)	0.0299 (4)	
O3	−0.2382 (2)	0.5471 (3)	0.01447 (13)	0.0319 (4)	
O5	0.0491 (3)	0.2401 (3)	−0.06531 (17)	0.0444 (5)	
H51	0.121 (4)	0.161 (4)	−0.043 (3)	0.044 (10)*	
H31	−0.277 (5)	0.650 (4)	−0.005 (3)	0.044 (10)*	
H42	0.043 (4)	0.751 (4)	−0.123 (2)	0.039 (9)*	
H52	−0.007 (5)	0.177 (7)	−0.100 (3)	0.073 (14)*	
H41	0.192 (4)	0.631 (5)	−0.126 (2)	0.040 (8)*	
H32	−0.275 (6)	0.554 (7)	0.0665 (16)	0.055 (12)*	

### Atomic displacement parameters (Å^2^)

**Table:**

	*U*^11^	*U*^22^	*U*^33^	*U*^12^	*U*^13^	*U*^23^
O2	0.0241 (7)	0.0453 (9)	0.0177 (6)	0.0001 (6)	−0.0027 (6)	0.0022 (6)
O1	0.0169 (7)	0.0595 (11)	0.0230 (8)	0.0000 (6)	0.0001 (6)	0.0051 (7)
N2	0.0198 (8)	0.0599 (13)	0.0203 (8)	0.0004 (8)	−0.0028 (8)	0.0006 (8)
C4	0.0149 (7)	0.0327 (10)	0.0176 (9)	0.0008 (6)	−0.0011 (7)	0.0013 (7)
N1	0.0162 (7)	0.0465 (11)	0.0236 (10)	0.0002 (6)	−0.0010 (7)	0.0021 (7)
C6	0.0160 (7)	0.0279 (8)	0.0203 (9)	0.0007 (6)	−0.0023 (7)	0.0007 (7)
C3	0.0186 (9)	0.0639 (15)	0.0180 (11)	0.0019 (9)	−0.0011 (8)	0.0004 (10)
C5	0.0175 (8)	0.0458 (12)	0.0210 (9)	0.0004 (8)	−0.0010 (8)	0.0012 (8)
Li1	0.0259 (15)	0.0370 (18)	0.0270 (18)	0.0004 (14)	0.0000 (16)	0.0022 (15)
O4	0.0282 (7)	0.0381 (9)	0.0235 (6)	0.0039 (6)	0.0062 (7)	0.0043 (6)
O3	0.0277 (7)	0.0433 (9)	0.0248 (8)	0.0045 (6)	0.0015 (6)	0.0026 (8)
O5	0.0602 (13)	0.0355 (9)	0.0376 (9)	0.0057 (9)	−0.0118 (10)	−0.0080 (8)

### Geometric parameters (Å, º)

**Table:**

O2—C6	1.274 (3)	C5—H5	0.9300
O1—C6	1.240 (3)	Li1—O3	1.905 (5)
N2—C3	1.331 (3)	Li1—O5	1.909 (4)
N2—N1	1.350 (3)	Li1—O4	1.960 (5)
Li1—N2	2.053 (5)	O4—H42	0.835 (19)
C4—C5	1.379 (3)	O4—H41	0.813 (19)
C4—C3	1.398 (3)	O3—H31	0.823 (19)
C4—C6	1.478 (3)	O3—H32	0.84 (2)
N1—C5	1.334 (3)	O5—H51	0.829 (19)
N1—H1	0.8600	O5—H52	0.80 (2)
C3—H3	0.9300		
			
C3—N2—N1	104.39 (18)	C4—C5—H5	126.5
C3—N2—Li1	125.9 (2)	O3—Li1—O5	115.6 (2)
N1—N2—Li1	129.66 (19)	O3—Li1—O4	109.1 (2)
C5—C4—C3	104.33 (18)	O5—Li1—O4	104.9 (2)
C5—C4—C6	128.0 (2)	O3—Li1—N2	107.0 (2)
C3—C4—C6	127.69 (19)	O5—Li1—N2	109.3 (2)
C5—N1—N2	112.55 (18)	O4—Li1—N2	110.9 (2)
C5—N1—H1	123.7	Li1—O4—H42	124 (2)
N2—N1—H1	123.7	Li1—O4—H41	114 (3)
O1—C6—O2	124.3 (2)	H42—O4—H41	112 (4)
O1—C6—C4	119.05 (19)	Li1—O3—H31	117 (3)
O2—C6—C4	116.66 (19)	Li1—O3—H32	121 (3)
N2—C3—C4	111.67 (19)	H31—O3—H32	100 (4)
N2—C3—H3	124.2	Li1—O5—H51	121 (3)
C4—C3—H3	124.2	Li1—O5—H52	134 (4)
N1—C5—C4	107.1 (2)	H51—O5—H52	103 (5)
N1—C5—H5	126.5		

### Hydrogen-bond geometry (Å, º)

**Table:**

*D*—H···*A*	*D*—H	H···*A*	*D*···*A*	*D*—H···*A*
O3—H32···O1^i^	0.84 (2)	1.87 (3)	2.666 (3)	159 (4)
O4—H41···O2^ii^	0.81 (2)	1.88 (2)	2.684 (2)	171 (4)
O5—H52···O2^iii^	0.80 (2)	2.04 (3)	2.813 (3)	163 (5)
O4—H42···O2^iv^	0.84 (2)	1.94 (2)	2.770 (3)	174 (4)
O3—H31···O4^v^	0.82 (2)	2.05 (3)	2.820 (3)	156 (4)
O5—H51···O3^vi^	0.83 (2)	1.98 (2)	2.804 (3)	172 (4)
N1—H1···O1^i^	0.86	1.96	2.776 (3)	159

Symmetry codes: (i) *x*−1, *y*, *z*; (ii) −*x*+1, −*y*+1, *z*−1/2; (iii) −*x*+1/2, *y*−1/2, *z*−1/2; (iv) −*x*+1/2, *y*+1/2, *z*−1/2; (v) *x*−1/2, −*y*+3/2, *z*; (vi) *x*+1/2, −*y*+1/2, *z*.
